# Draft Genome Sequences of Six Yersinia kristensenii Strains

**DOI:** 10.1128/MRA.01063-21

**Published:** 2022-01-06

**Authors:** Angelina A. Kislichkina, Mikhail E. Platonov, Yury P. Skryabin, Angelika A. Sizova, Lidia A. Shishkina, Elena V. Galkina, Alexandr G. Bogun, Svetlana V. Dentovskaya

**Affiliations:** a State Research Center for Applied Microbiology and Biotechnology, Obolensk, Russia; b Pushchino State Institute of Natural Science, Pushchino, Russia; University of Maryland School of Medicine

## Abstract

Yersinia kristensenii is one of the Yersinia enterocolitica-like bacterial species, which are considered nonpathogenic to humans. In this work, we reported the draft genome sequences of six Yersinia kristensenii strains. These draft genomes will help to better characterize Yersinia kristensenii at the genomic level.

## ANNOUNCEMENT

The genus *Yersinia* includes Gram-negative rod-shaped facultative anaerobes belonging to the *Enterobacteriaceae* family (1). Three of its species, Yersinia pestis, Yersinia pseudotuberculosis, and Yersinia enterocolitica, are well known for their pathogenicity in humans (2). The remaining species, which are considered nonpathogenic to humans, have been investigated significantly less (3). One of these species, Yersinia kristensenii, was derived from Y. enterocolitica and defined as a new species by Bercovier and colleagues in 1980, based on DNA-DNA hybridizations and biochemical characteristics (4). Trehalose-positive, sucrose-negative bacteria belonging to this species have frequently been isolated from a variety of environmental samples (such as freshwater and soil samples), foods, animals (including horses, sheep, and monkeys), and healthy and sick humans worldwide (3, 5).

In this report, we announce draft genome sequences of six Y. kristensenii strains stored in the microorganism collection of the State Research Center for Applied Microbiology and Biotechnology (SRCAMB). Bacteria were originally identified as Yersinia enterocolitica-like, as confirmed with microscopic examinations and biochemical identification tests. Before whole-genome sequencing, their species identifications were updated by matrix-assisted laser desorption ionization (MALDI) Biotyper identification and biochemical identification tests.

The stocks of Y. kristensenii strains were stored at −70°C in cryoprotective medium. Bacteria were grown at 37°C on nutrient medium 1 (Obolensk, Russia). DNA from each strain was extracted using the DNA minikit (BioFact, Daejeon, Republic of Korea) following the manufacturer’s instructions. For Y. kristensenii strain SCPM-O-B-7606, whole-genome sequencing was performed using the Ion 318 chip kit and 400-bp chemistry on the Ion Torrent PGM platform. Whole-genome sequencing of the other Y. kristensenii strains was performed using the Nextera DNA library preparation kit and MiSeq reagent kit v3 on the Illumina MiSeq platform. Reads were *de novo* assembled using two assemblers with default settings, which included primary filtering and quality control, i.e., SPAdes v3.9.0 for Y. kristensenii strains SCPM-O-B-7606, SCPM-O-B-3969, SCPM-O-B-7953, and SCPM-O-B-8071 and Unicycler v0.4.7 for Y. kristensenii strains SCPM-O-B-7961 and SCPM-O-B-7962 (6, 7).

The draft genome sizes ranged from 4.47 to 4.77 Mb, with the GC content for Y. kristensenii strain SCPM-O-B-8071 being 47.7% and that for the other strains being 47.5%. The number of contigs per assembly for each isolate ranged from 17 to 264. The final assemblies were annotated using the NCBI Prokaryotic Genome Annotation Pipeline (PGAP) (8). The annotation method and annotation software version used are listed in Table 1. The genomes contain 4,001 to 4,593 coding sequences (CDSs).

**TABLE 1:** Genome assembly details and statistics

Strain name	Isolation source	SRA accession no. (raw data)	Sequencing platform	Avg read length (bp)	No. of reads	Read length (bp)	Genome size (bp)	Coverage (×)	No. of contigs	No. of CDSs	GC content (%)	*N*_50_ (bp)	GenBank accession no.	NCBI PGAP analysis	
														Annotation method	Annotation software version
SCPM-O-B-3969	Unknown	SRR6206395	Illumina MiSeq	444	224,788	100,000,842	4,530,867	21	61	4,214	47.5	155,225	PGWV00000000	Best-placed reference protein set; GeneMarkS+	4.3
SCPM-O-B-7606	*Microtus arvalis*	SRR5298263	Ion Torrent PGM	210	394,287	83,180,127	4,737,791	18.0	264	4,593	47.5	42,862	MWTL00000000	Best-placed reference protein set; GeneMarkS+	4.1
SCPM-O-B-7953	*Microtus arvalis*	SRR6206394	Illumina MiSeq	454	247,148	112,268,430	4,513,011	24	45	4,207	47.5	242,455	PGLT00000000	Best-placed reference protein set; GeneMarkS+	3.2
SCPM-O-B-7961	*Microtus arvalis*	SRR11355546	Illumina MiSeq	509	433,421	220,848,897	4,472,460	49.4	29	4,005	47.5	612,624	JAASAM000000000	Best-placed reference protein set; GeneMarkS-2+	4.11
SCPM-O-B-7962	Flush from potatoes	SRR11355545	Illumina MiSeq	575	275,355	158,351,826	4,467,037	35.4	17	4,001	47.5	651,285	JAASAL000000000	Best-placed reference protein set; GeneMarkS-2+	4.11
SCPM-O-B-8071	Unknown	SRR6206392	Illumina MiSeq	412	221,281	91,214,041	4,768,474	19	104	4,479	47.7	89,657	PEHL00000000	Best-placed reference protein set; GeneMarkS+	4.2

### Data availability.

The genome sequences and sequence reads were deposited in the GenBank database under the accession numbers listed in Table 1.
